# Supplementary figures and images for: Transcriptome analysis of chloride intracellular channel knockdown in Drosophila identifies oxidation-reduction function as possible mechanism of altered sensitivity to ethanol sedation
Source: PLoS One. 2021 Jul 6;16(7):e0246224. doi: 10.1371/journal.pone.0246224 (PMC8259981; doi:10.1371/journal.pone.0246224)

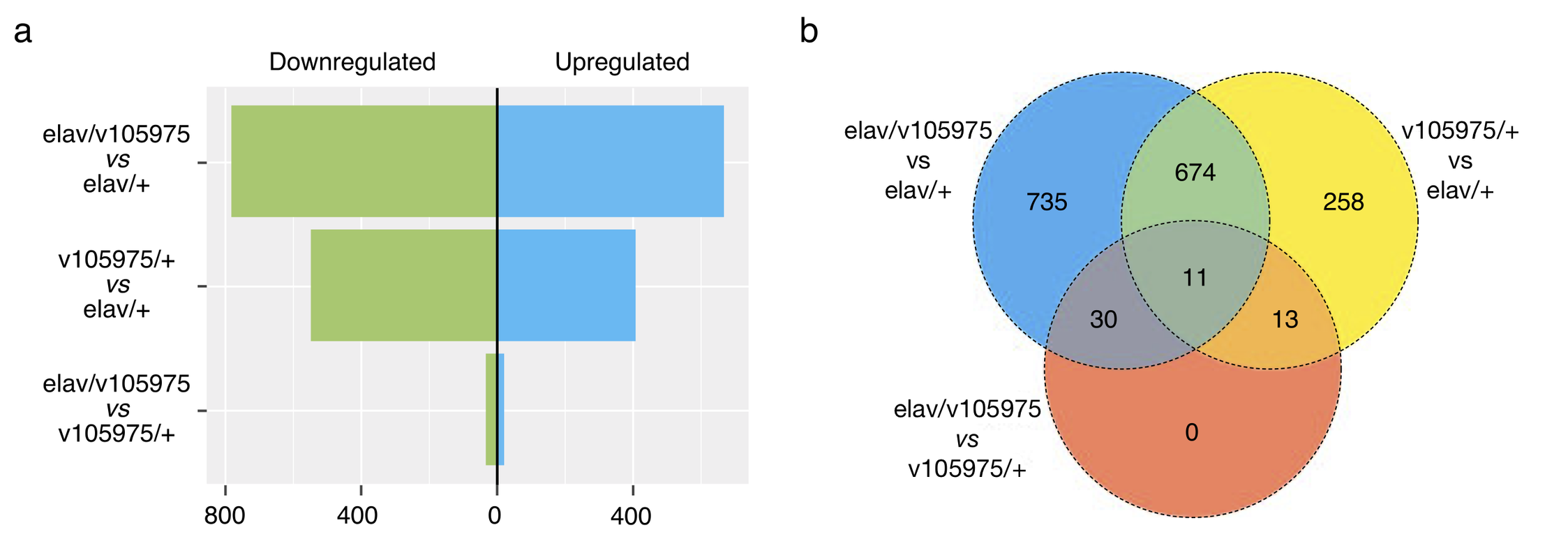

Supplement: S1 Fig — (a) Differentially regulated genes (FDR ≤ 0.05) for each possible fly strain contrast. (b) Venn diagram analysis of genes differentially expressed between knockdown (elav/v105975) or RNAi-only control (v105975) vs Gal4-only control (elav/+) shows substantial overlap of gene sets. (TIF) [file pone.0246224.s001.tif]

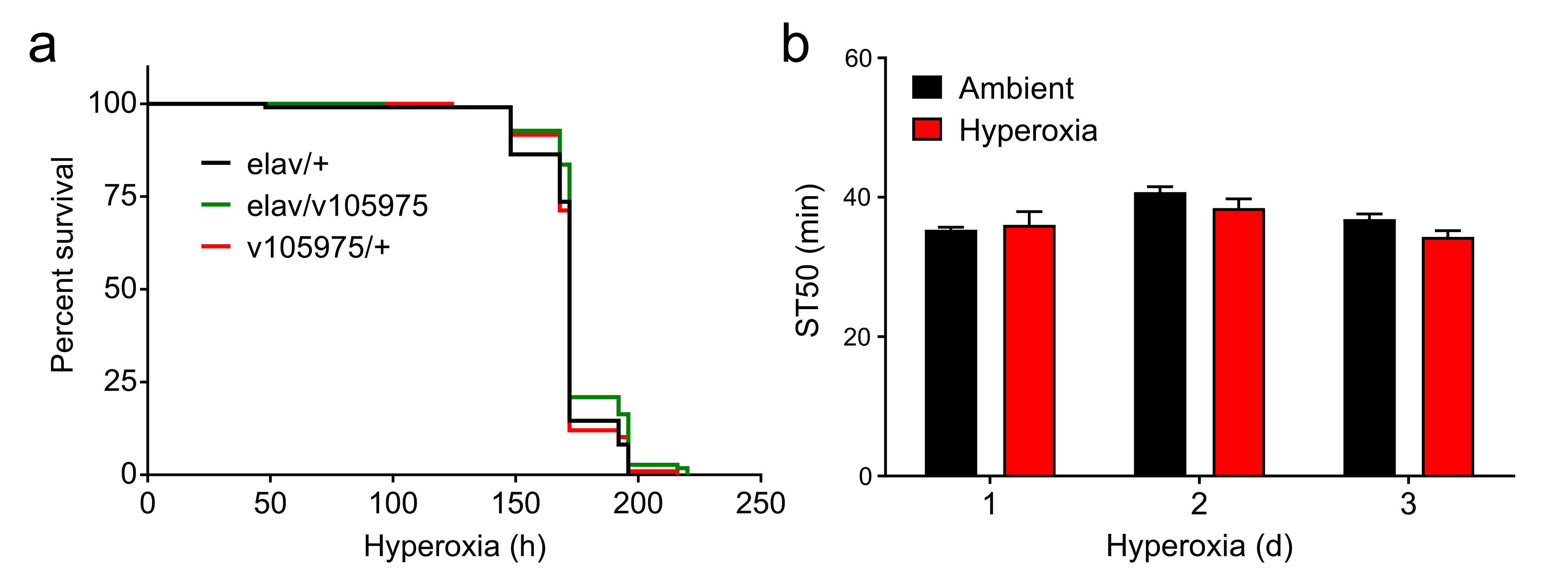

Supplement: S2 Fig — (a) Survival analysis for flies exposed to continuous hyperoxia grouped by strain. (b) Ethanol sedation times for wild-type control flies under ambient and hyperoxic conditions for 3 days. (TIF) [file pone.0246224.s002.tif]
